# Supplementary material for: Early antiretroviral therapy favors post-treatment SIV control associated with the expansion of enhanced memory CD8+ T-cells
Source: Nat Commun. 2024 Jan 11;15:178. doi: 10.1038/s41467-023-44389-3 (PMC10784587; doi:10.1038/s41467-023-44389-3)
Supplement: Supplementary file 1 — Supplementary Information [file 41467_2023_44389_MOESM1_ESM.pdf]

SUPPLEMENTARY MATERIAL

Table S1. Characteristics of Cynomolgus macaques included in the study.

ID	Experimental group	Study phase	Outcome at euthanasia	Days post-ATI at euthanasia	MHC Haplotype	Age at inclusion (years)	Weight at inclusion (kg)
BA922I	ART W4	pVISCONTI-1	PTC	380	M1/M3	5.8	5.4
CA706F	ART W4	pVISCONTI-1	PTC	391	M5/M4M1M2	4.1	4.4
BA777K	ART W4	pVISCONTI-1	Non-PTC	247	M2/M1	4.8	6.7
BA736J	ART W4	pVISCONTI-1	PTC	394	M1M2M1/M4	5.2	5.5
CCB065	ART W4	pVISCONTI-1	PTC	387	M3/M4	4.2	6.4
BB123J	ART W4	pVISCONTI-1	PTC	384	M2M1/M3	5.2	5.8
BB799G	ART W4	pVISCONTI-2	PTC	374	M3M5/M2M3	4.3	6.5
CB806C	ART W4	pVISCONTI-2	PTC	385	M1M5M3/M3	4.7	6.54
BB103G	ART W4	pVISCONTI-2	Non-PTC	371	M4/M2	7.2	7.9
CB296A	ART W4	pVISCONTI-2	Rapid progressor	N/A	M4/M1	6.7	7.25
BA797I	ART W4	pVISCONTI-2	PTC	378	M1/M3M4	7	7.65
BB9I	ART W4	pVISCONTI-2	PTC	381	M1/M2	6.7	9.05
CCE007	ART W24	pVISCONTI-1	Non-PTC	209	M1/M3	4	4.7
CCB063	ART W24	pVISCONTI-1	Non-PTC	155	M1/M2M5	4.2	6.1
BA912K	ART W24	pVISCONTI-1	Non-PTC	195	M1/M2	4.8	5
BA987H	ART W24	pVISCONTI-1	PTC	295	M4/M3	5.1	5.5
CCB114	ART W24	pVISCONTI-1	Non-PTC	191	M3M4/M2	4.2	4.6
BB425F	ART W24	pVISCONTI-1	Non-PTC	168	M1/M2M5	5.1	5.8
CCB090	ART W24	pVISCONTI-2	PTC	237	M3M1M3/M5	4.6	5.2
BA979I	ART W24	pVISCONTI-2	Natural controller	N/A	M4/M2	6.6	8.55
BB340E	ART W24	pVISCONTI-2	Non-PTC	226	M3M1/M4	6.5	8.5
BA922J	ART W24	pVISCONTI-2	Non-PTC	240	M1/M3	4.5	7.3
BA733K	ART W24	pVISCONTI-2	Non-PTC	244	M1M4/M2	6.5	9.67
BA878K	ART W24	pVISCONTI-2	Non-PTC	247	M3/M2	5.5	6.6
CCA032	non-treated	pVISCONTI-0	Progressor	N/A	M3M2/M3	3.3	3.8
CCA033	non-treated	pVISCONTI-0	Progressor	N/A	M5M1/M4M1	3.3	3.2
CCA035	non-treated	pVISCONTI-0	Progressor	N/A	M3/M1M4	3.3	4.8
CCA100	non-treated	pVISCONTI-0	Progressor	N/A	M5/M1	3.3	4.8
CCB117	non-treated	pVISCONTI-0	Progressor	N/A	M1/M2	3.2	3.9
CCC039	non-treated	pVISCONTI-0	Progressor	N/A	M4/M3	3.1	3.8
CCB028	non-treated	pVISCONTI-3	Progressor	N/A	M1/M1	3.9	4.45
CCB070	non-treated	pVISCONTI-3	Progressor	N/A	M3/M3M4	3.9	4.61
CCC066	non-treated	pVISCONTI-3	Progressor	N/A	M1/M2	3.8	5.74
CCD021	non-treated	pVISCONTI-3	Rapid progressor	N/A	M4/M2	3.7	5.13
CBK061	non-treated	pVISCONTI-3	Progressor	N/A	M4M1M2/M2M3	4	4.4
CDJ017	non-treated	pVISCONTI-6	Progressor	N/A	M1/M2M5	5.3	5.51
CDJ031	non-treated	pVISCONTI-6	Progressor	N/A	M2/M3	5.3	7.67
CDJ036	non-treated	pVISCONTI-6	Progressor	N/A	M4/M1M4	5.3	7.17

CDJ052	non-treated	pVISCONTI-6	Natural controller	N/A	M2/M1M5	5.3	7.25
CDJ053	non-treated	pVISCONTI-6	Progressor	N/A	M1/M1M2	5.3	7.69
CDK079	non-treated	pVISCONTI-6	Progressor	N/A	M2/M5	5.2	5.71

Post-treatment controller, PTC ; Non-post-treatment controller, non-PTC; not-applicable, N/A

Table S2. List of optimal peptides used to evaluate $CD8^+$ T-cell responses.

SIV protein	Amino acid positions	Length (amino acids)	Amino acid sequence	Primary restricting haplotype	Restricting Molecule*
Gag	28–37	10	KYMLKHVVWA	M3	Mafa B*011:01
Gag	54–63	10	KEGCQKILSV	M3	Mafa B*075:01
Gag	146–154	9	HLPLSPRTL	M3	Mafa B*075:01
Gag	192–200	9	NCVGDHQAA	M1	Mafa B*104:01
Gag	221–229	9	PAPQQGQLR	M3	Mafa B*075:01
Gag	386–394	9	GPRKPIKCW	M3	Mafa A1*063:02
Gag	459–467	9	TAPPEDPAV	M3	Mafa B*075:01
Pol	592–600	9	QVPKFHLPV	M1,M2, M3	Mafa A4*01:01
Env	260–268	9	VSSCTRMME	M3	Mafa B*075:01
Env	338–346	9	RPKQAWCWF	M3	Mafa A1*063:02
Env	504–512	9	PIGLAPTDV	M3	Mafa B*075:01
Env	620–628	9	TVPWPNASL	M3	Mafa B*075:01
Tat	42–49	8	QLYRPLEA	M3	Mafa B1*075:01
Tat	59–67	9	CCYHCQFCF	M3	Mafa A1*063:02
Rev	26–34	9	YPTGPGTAN	M3	Mafa A1*063:02
Rev	59–68	10	SFPDPPTDTP	M3	Mafa B*075:01
Nef	103–111	9	RPKVPLRTM	M3	Mafa A1*063:02
Nef	103–112	10	RPKVPLRTMS	M1,M2	Mafa A1*063:01
Nef	194–203	10	LMHPAQTSQW	M3	Mafa B*011:01
Nef	196–203	8	HPAQTSQW	M1, M2, M3	Mafa A1*063
Nef	238–248	11	GLSEEEVRRRL	M3	Mafa B*011:01
Vif	155–163	9	VVSDVRSQGE	M3	Mafa B*011:01

*M1, M2 and M3 haplotypes transcribe strongly similar Mafa-A1*063 alleles [69]10.1007/s00251-010-0481-9) . The optimal peptides described to be restricted by Mafa A1*063:01 and Mafa A1*063:02 in our table could virtually be recognized by the M1, M2 and M3 haplotypes.

Figure S1

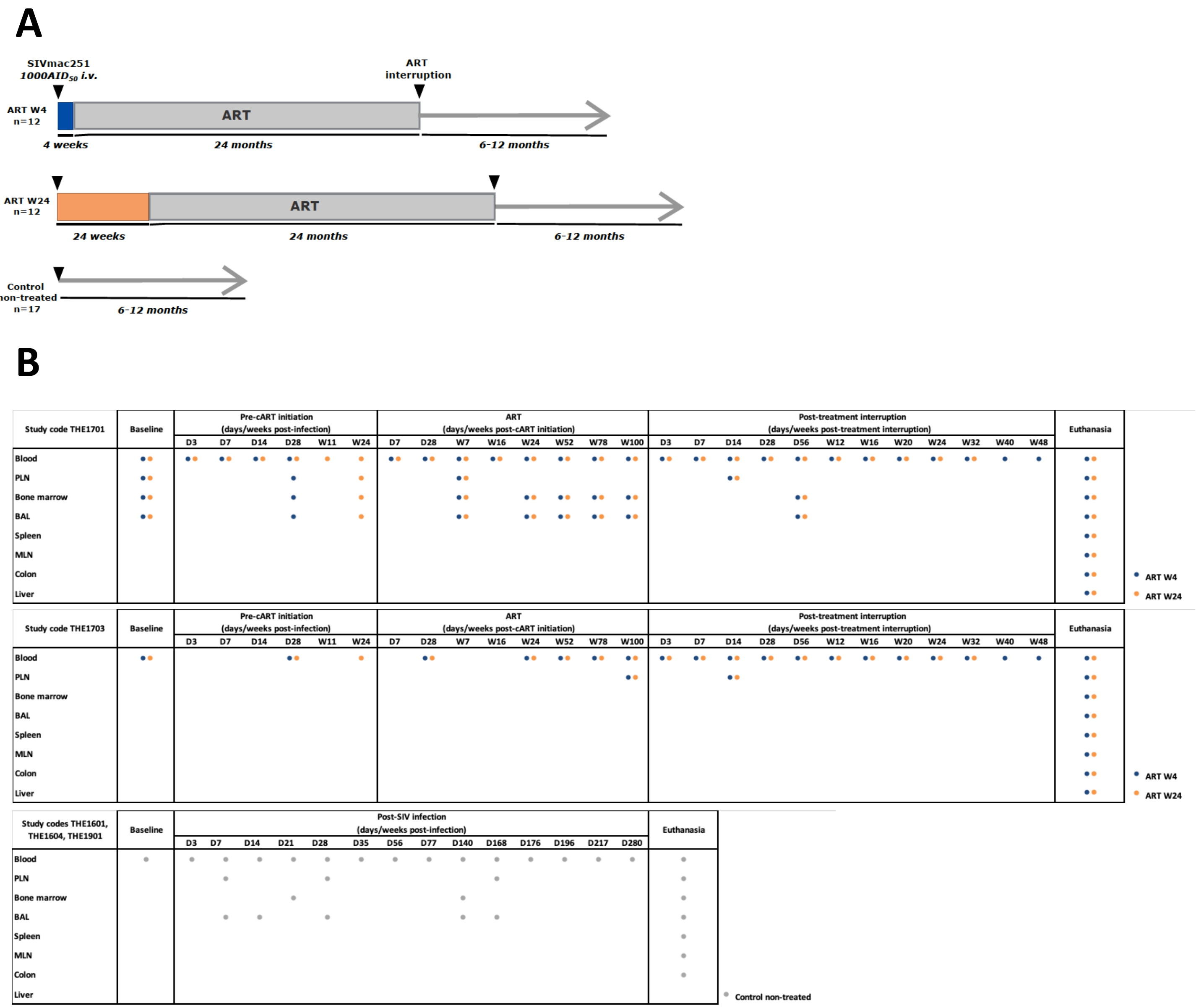

Figure S1. Study design. A) Schematic representation of the pVISCONTI study design. CyMs were infected with 1000 AID_{50} of $SIVmac_{251}$ intravenously (i.v.). Twelve animals received combination antiretroviral therapy (FTC, DTG and TDF) starting four weeks post-infection (W4-treated, blue bar) and twelve started ART at 24 weeks post-infection (W24-treated, orange bar). Animals received intradermal ART daily across 24 months and then underwent treatment interruption. Following treatment interruption these CyMs were closely monitored for 24 to 48 weeks before euthanasia. Seventeen animals remained untreated (grey arrow) and were followed up for 24 to 48 weeks for comparison. **B**) The pVISCONTI study was designed to be performed in several phases, i.e., CyMs were monitored as independent experiments named pVISCONTI-1 (n=6 W4-treated and n=6 W24-treated), pVISCONTI-2 (n=6 W4-treated and n=6 W24-treated), pVISCONTI-0 (n=6 untreated), pVISCONTI-3 (n=5 untreated), pVISCONTI-6 (n=6 untreated). The time points analyzed and the samplings corresponding to each time point are indicated by dots colored accordingly (W4-treated = blue dots; W24-treated = orange dots; untreated = grey dots).

Figure S2

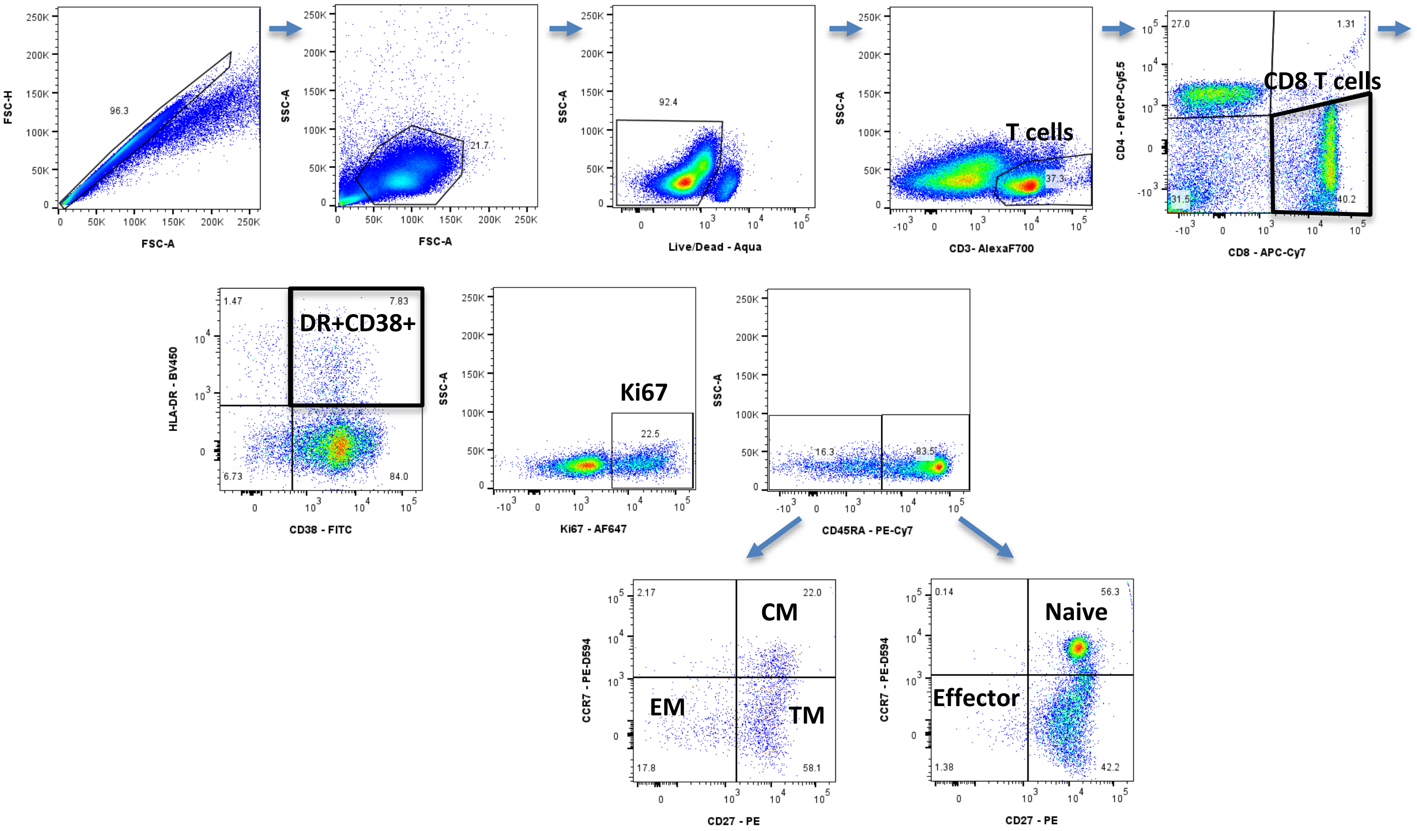

Figure S2. Flow cytometric gating strategy used to analyze the differentiation and activation phenotype of CD8+ T-cells. Results are depicted as standard pseudocolor dot plots for samples from one macaque. Central memory (CM), transitional memory (TM), effector memory (EM).

Figure S3

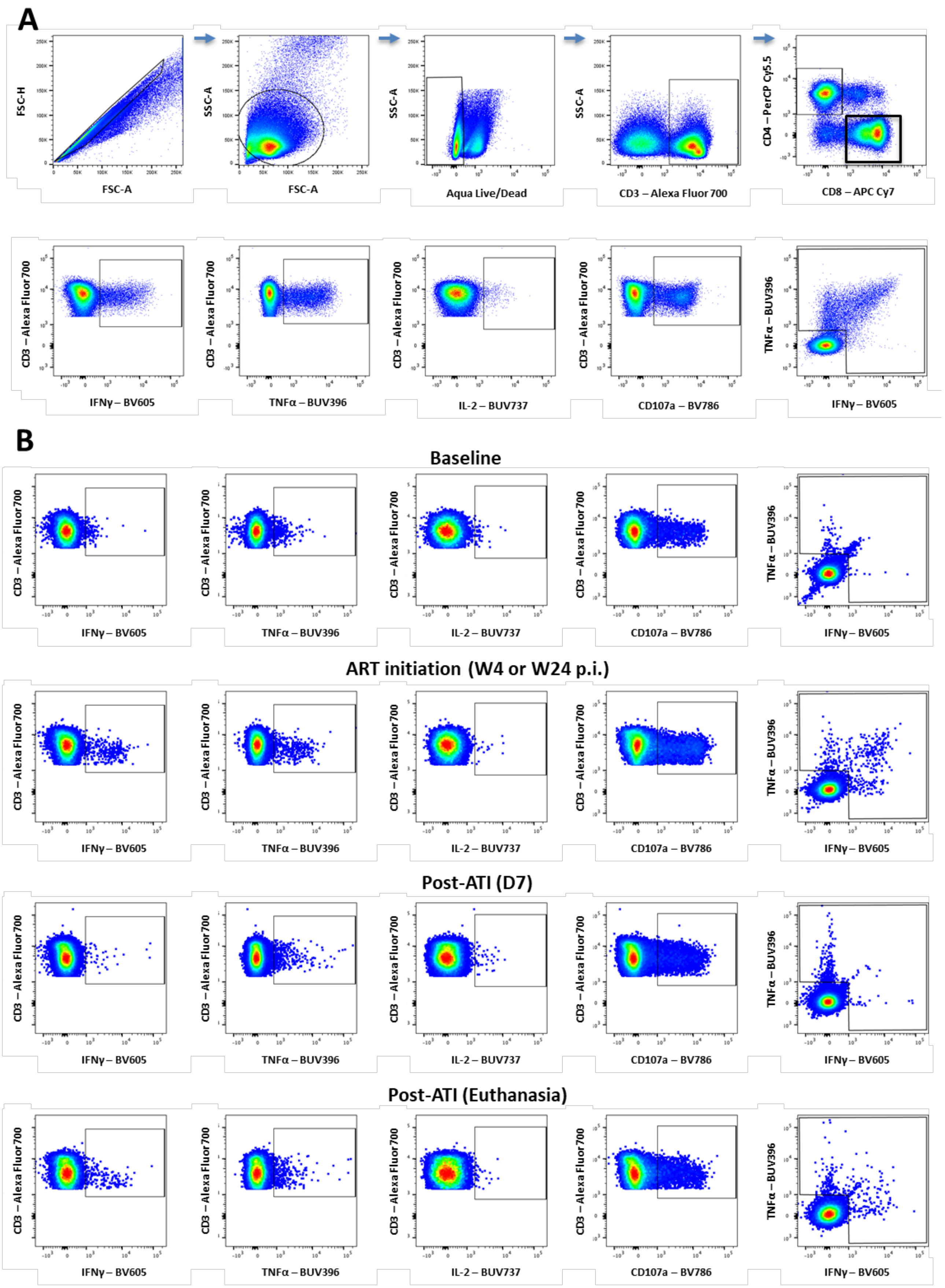

Figure S3. Flow cytometric gating strategy for the analysis of antigen specific cells. A) Flow cytometric gating strategy used to analyze cytokine production by PBMCs via intracellular staining. Concanavalin A stimulation is shown as an example. **B**) Representative example of cytokine production by PBMCs of a CyM (CA706F) at baseline, at the time of ART initiation, and post-ATI (day 7 post-ATI and at euthanasia) stimulated with the pool of optimal SIV peptides. Results are depicted as standard pseudocolor dot plots.

Figure S4

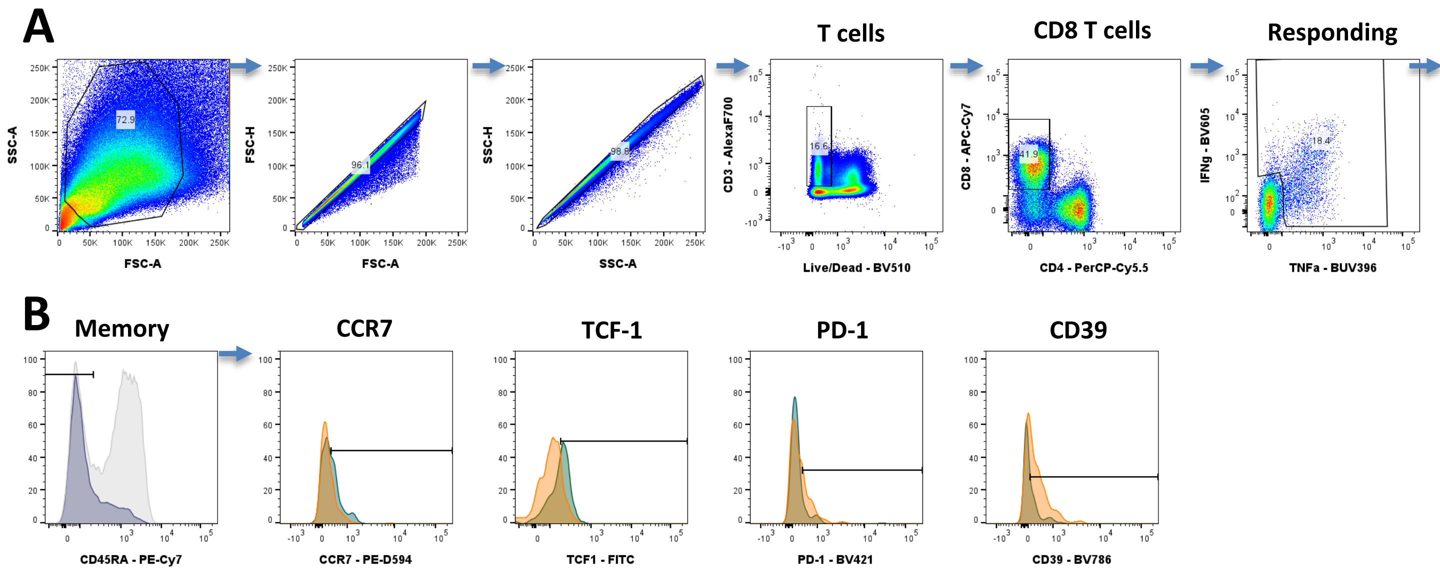

Figure S4. Gating strategy for memory CD8+ T cells. (A) Flow cytometric gating strategy used to analyze the memory profile of CD8+ T-cells producing IFNγ and/or TNFα in response to stimulation. Results are depicted as standard pseudocolor dot plots for samples from one macaque. **(B)** Comparison of CCR7, TCF-1, PD-1 and CD39 expression on SIV-specific memory CD8+ T cells from one W4-treated (green) and one W24-treated (orange) animal.

Figure S5

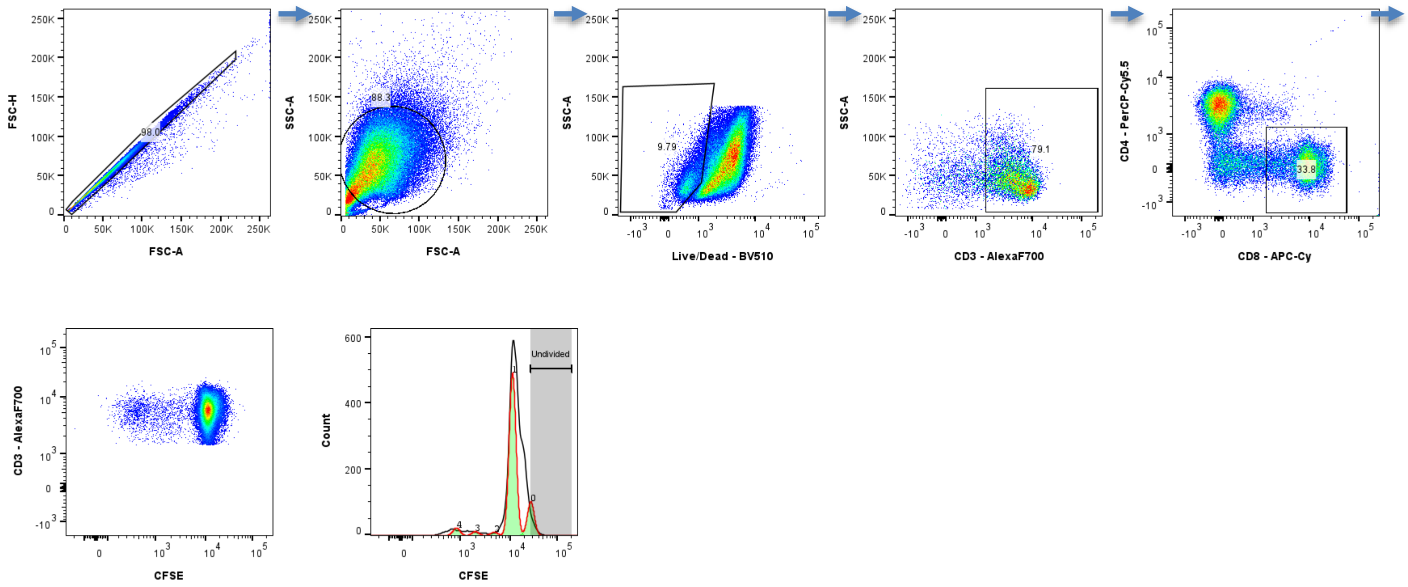

Figure S5. Flow cytometric gating strategy used to analyze the proliferation potential of CD8+ T-cells after stimulation with a pool of optimal SIV peptides. Results are depicted as standard pseudocolor dot plots for samples from one macaque and the correspondent proliferation modeling performed by FlowJo algorithm.

Figure S6

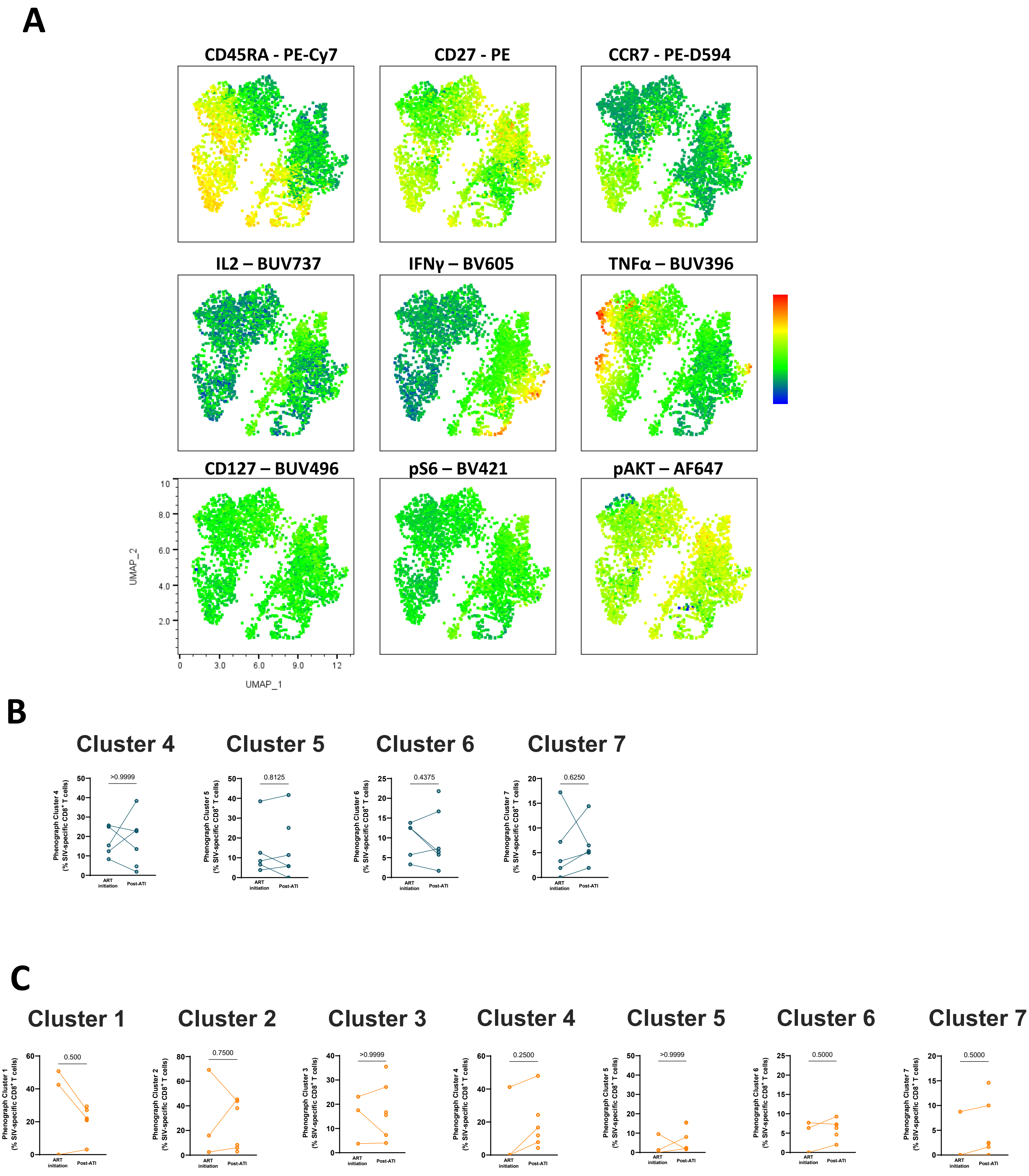

Figure S6. Analysis of SIV specific CD8+ T cells. (A) UMAP plot of 2979 SIV-specific CD8$^+$ T cells from the blood of W4- and W24-treated CyMs at the time of ART initiation and post-ATI showing the relative expression of the markers that were used for the unsupervised identification of T cell clusters by PhenoGraph. Dynamics of the phenotypically distinct clusters of SIV-specific CD8$^+$ T cells defined by PhenoGraph at the time of ART initiation and post-ATI in (**B**) W4-treated (clusters 4 to 7)(n=6) and (**C**) W24-treated (clusters 1 to 7) CyMs (n=6). Individual data are shown. Two-sided Wilcoxon matched-pairs rank test. Source data are provided as a Source Data file.

Figure S7

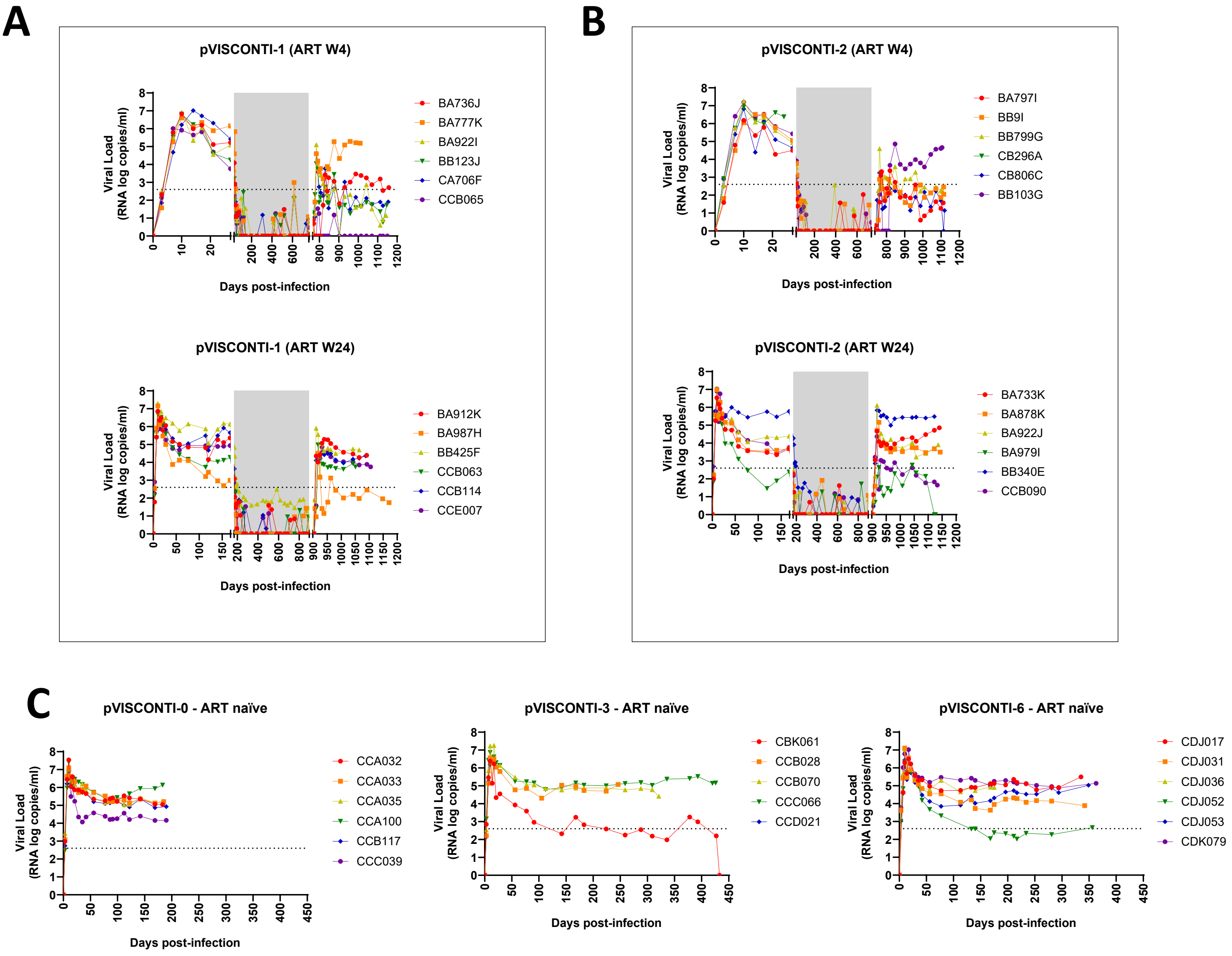

Figure S7. Individual plasma viral load kinetics along the follow up for the independent groups analyzed in the pVISCONTI study. pVISCONTI-1 **(A)** and pVISCONTI-2 **(B)** were independent experiments comparing W4-treated and W24-treated macaques (n=6 per group). **(C)** pVISCONTI-0 (n=6), pVISCONTI-3 (n=5) and pVISCONTI-6 (n=6) were experiments where infected macaques did not receive antiretroviral treatment. Individual data are shown. Source data are provided as a Source Data file.

Figure S8

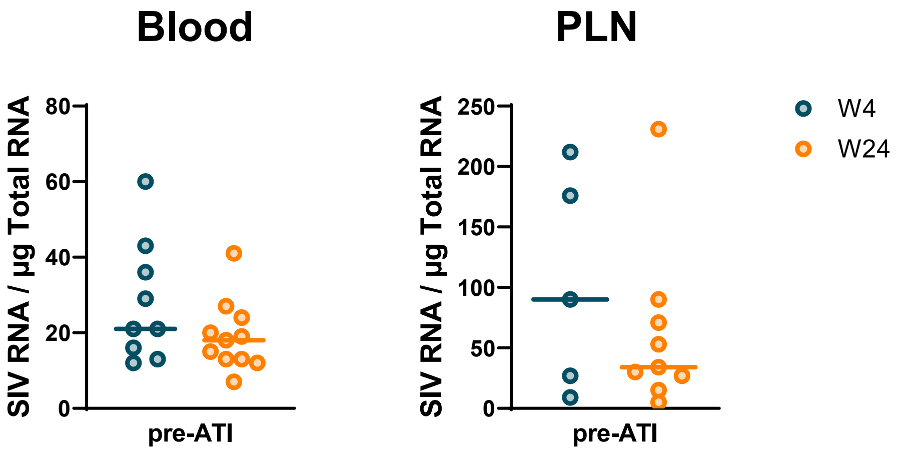

Figure S8. Cell associated viral RNA. Comparison of cell-associated SIV RNA in blood and PLN of W4- (n=11) and W24-treated CyMs (n=11) at the at the time of treatment interruption. Individual values and medians are shown. Two-sided Mann–Whitney U test were used, no significant differences were found. Source data are provided as a Source Data file.

Figure S9

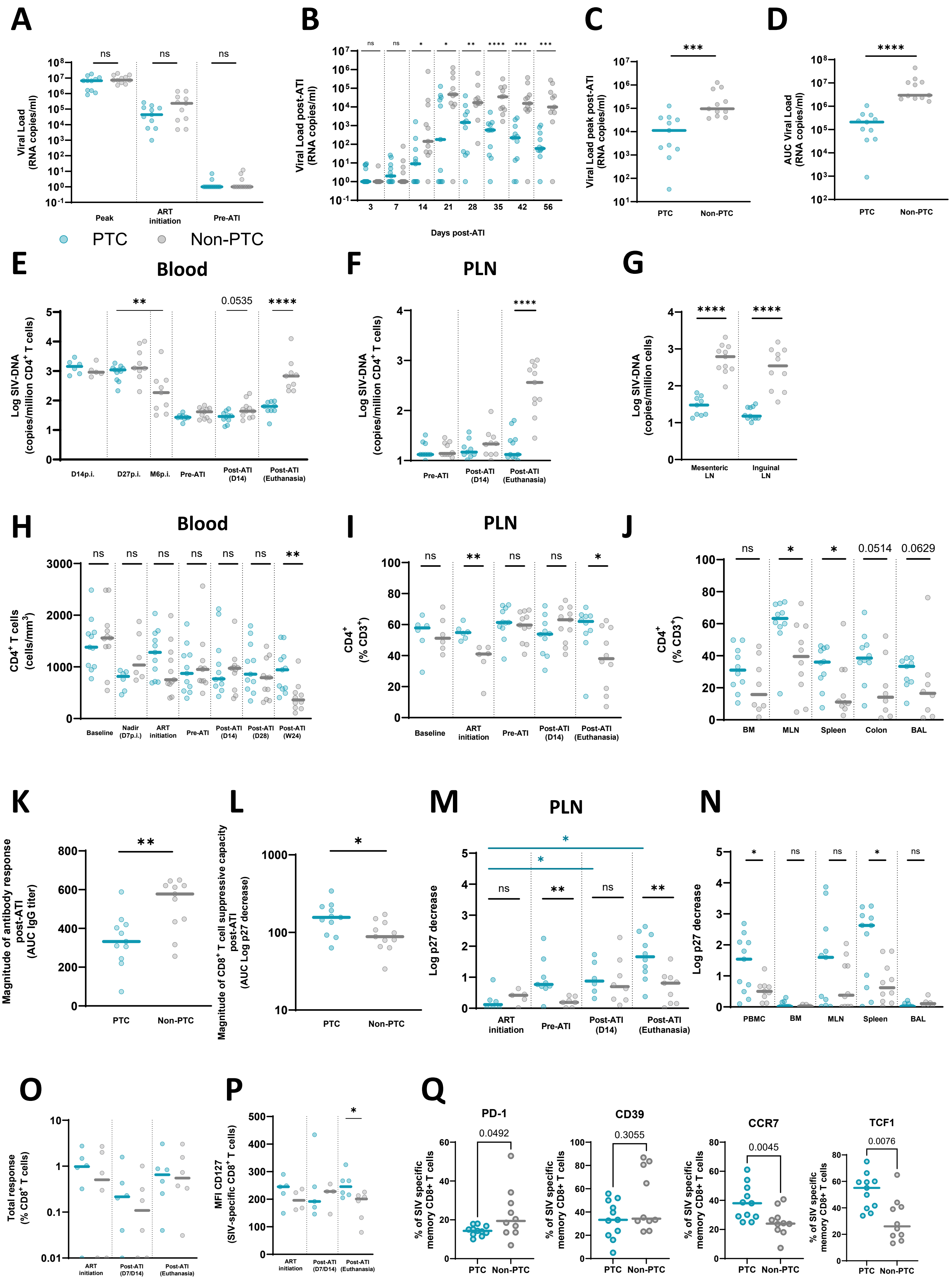

A
Viral Load (RNA copies/ml)
Peak
ART initiation
Pre-ATI
ns
PTC
Non-PTC
B
Viral Load post-ATI (RNA copies/ml)
Days post-ATI
C
Viral Load peak post-ATI (RNA copies/ml)
D
AUC Viral Load (RNA copies/ml)
E
Blood
Log SIV-DNA (copies/million CD4+ T cells)
D14p.i.
D27p.i.
M6p.i.
Post-ATI (D14)
Post-ATI (Euthanasia)
0.0535
F
PLN
G
Log SIV-DNA (copies/million cells)
Mesenteric LN
Inguinal LN
H
CD4+ T cells (cells/mm3)
Baseline
Nadir (D7p.i.)
Post-ATI (D28)
Post-ATI (W24)
I
CD4+ (% CD3+)
J
BM
MLN
Spleen
Colon
BAL
0.0514
0.0629
K
Magnitude of antibody response post-ATI (AUC IgG titer)
L
Magnitude of CD8+ T cell suppressive capacity post-ATI (AUC Log p27 decrease)
M
Log p27 decrease
N
PBMC
O
Total response (% CD8+ T cells)
Post-ATI (D7/D14)
P
MFI CD127 (SIV-specific CD8+ T cells)
Q
PD-1
CD39
CCR7
TCF1
% of SIV specific memory CD8+ T cells
0.0492
0.3055
0.0045
0.0076

Figure S9. Characteristics of animals when classified as post-treatment controllers or non post-treatment controllers. A) Comparison of pVL levels between post-treatment controllers (PTC, blue symbols) and non-controllers (non-PTC, grey symbols) CyMs at the peak (acute infection), at the time of ART initiation and prior to treatment interruption. **B**) Comparison of pVL levels between PTC and non-PTC CyMs at the early days following treatment interruption. The magnitude of plasma viral load post-ATI in PTC and non-PTC CyMs is indicated by (**C**) the viral load peak and by (**D**) the cumulative pVL post-ATI (area under the curve - AUC, considering all pVL measurements until 24 weeks post-ATI). Kinetics of SIV-DNA levels in (**E**) blood CD4, and in (**F**) PLN cells in PTC and non-PTC CyMs. **G**) Levels of SIV-DNA in mesenteric and inguinal lymph nodes at euthanasia. Results are expressed as copies SIV-DNA/million cells. Longitudinal evolution of $CD4^+$ T cell counts in (**H**) blood, and in (**I**) PLN in PTC and non-PTC CyMs. Results are shown as absolute $CD4^+$ T cell counts in blood and as proportion of $CD4^+$ T cells among $CD3^+$ lymphocytes in peripheral lymph nodes. (**J**) Proportion of $CD4^+$ T cells among $CD3^+$ lymphocytes in BM, MLN, spleen, colon mucosa and BAL at euthanasia. The magnitude of **K**) humoral response and **L**) $CD8^+$ T-cell-mediated SIV-suppressive activity in PTC and non-PTC CyMs post-ATI are indicated by the cumulative results post-ATI (area under the curve - AUC of all measurements until 24 weeks post-ATI). **M**) Kinetics of $CD8^+$ T cell-mediated SIV-suppressive activity in the PLN of PTC and non-PTC CyMs at the time of ART initiation (W4 or W24 p.i.), 14 days post-ATI and at euthanasia. **N**) $CD8^+$ T cell-mediated SIV-suppressive activity in PBMC, BM, MLN, spleen, and BAL at euthanasia. **O**) Total $CD8^+$ T cells SIV-specific response in the blood of PTC and non-PTC CyMs at the time of ART initiation and post-ATI. **P**) CD127 expression levels in SIV-specific $CD8^+$ T cells at the time of ART initiation and post-ATI. **Q**) Proportion of SIV-specific memory $CD8^+$ T cells expressing PD-1, CD39, CCR7 or TCF1 in the spleen at the end of the study. (**A-Q**) Individual values (n=6 or 11 per group) and medians are shown. *$p < 0.05$, **$p < 0.01$; ***$p < 0.001$; ****$p < 0.0001$; Two-sided Mann-Whitney U-test.

Figure S10

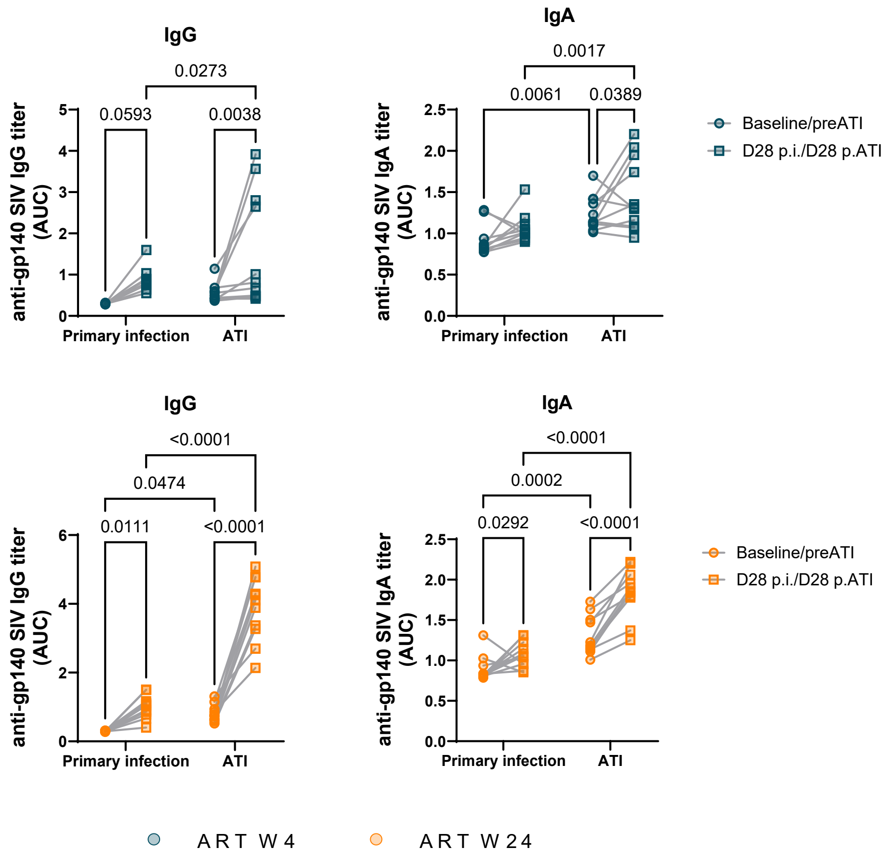

Figure S10. Increase in the anti-SIV antibody levels following primary infection or treatment interruption. Evolution of anti-gp140 IgG and IgA titers from baseline to 28 days p.i. and from time of ART interruption to 28 days post-ATI in W4- and W24-treated macaques (n=11 per group). Individual data are shown. Repeated measures 2-way ANOVA and Fisher's test for post-hoc analyses.

Figure S11

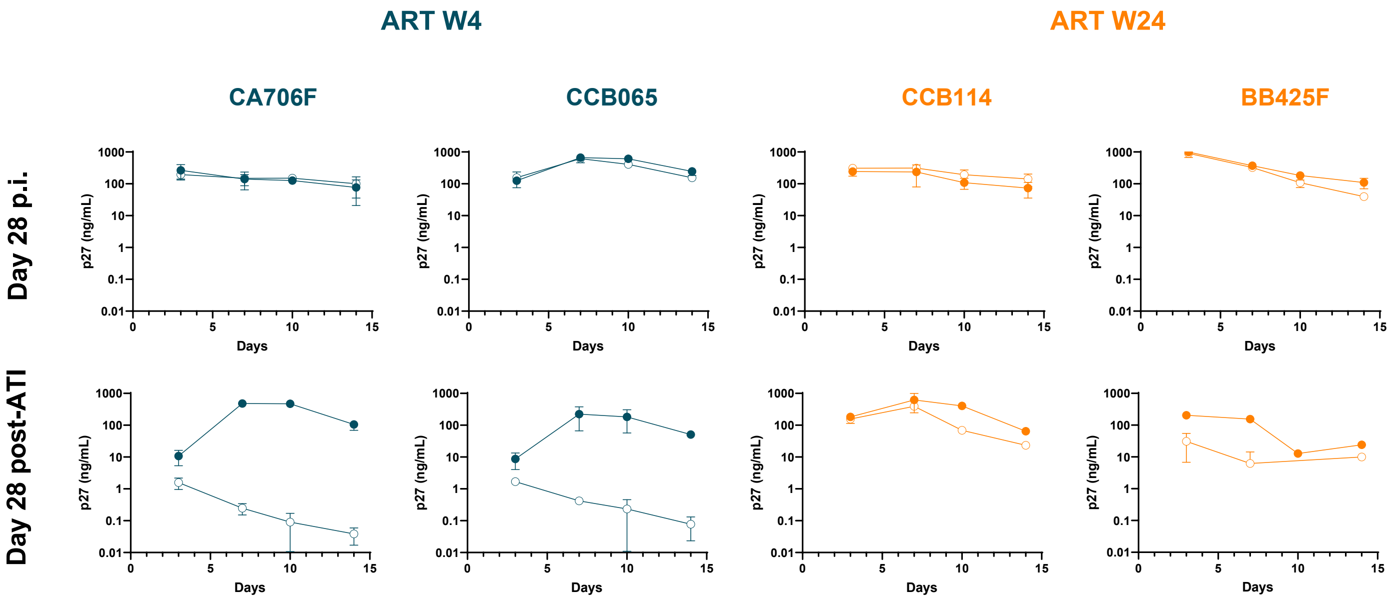

Figure S11. SIV suppression assay. Representative results obtained with the CD8⁺ T cell-mediated SIV-suppressive activity assay comparing blood samples from W4-treated and W24-treated CyMs at 28 days post-infection (top panels) and at 28 days post-ATI (bottom panels). The kinetics of SIV p27 production along 14 days of culture *in vitro* are presented. CD4⁺ T cells were cultured alone (filled dots) or cocultured with autologous CD8⁺ T cells (1:1 ratio) (empty dots). Mean and standard deviation for three replicates are shown.

Figure S12

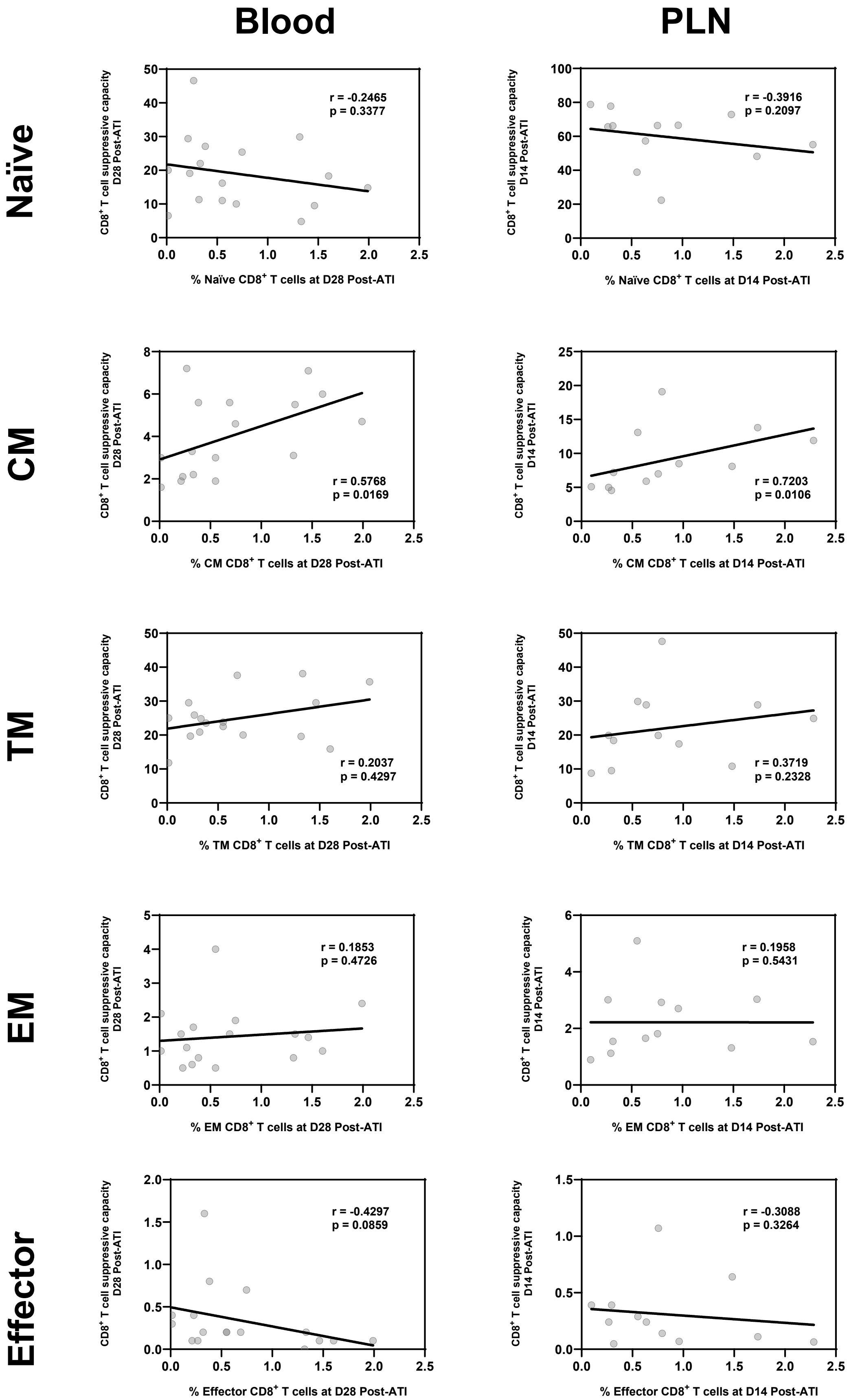

Figure S12 Correlations between CD8+ T cell subpopulations and SIV-suppressive capacity. Spearman correlation between the frequency of Naïve, Central memory (CM), Transitional memory (TM), Effector memory (EM) and Effector CD8$^+$ T cells, and the CD8$^+$ T cell mediated SIV-suppressive activity at 28 days post-ATI in blood (n=17 animals)(left panels) and at 14 days post-ATI in PLN (n=12 animals)(right panels). Linear regression (lines) and p are indicated for each comparison.
