# Supplementary material for: Early antiretroviral therapy favors post-treatment SIV control associated with the expansion of enhanced memory CD8+ T-cells
Source: Nat Commun. 2024 Jan 11;15:178. doi: 10.1038/s41467-023-44389-3 (PMC10784587; doi:10.1038/s41467-023-44389-3)
Supplement: Supplementary file 3 — Reporting Summary [file 41467_2023_44389_MOESM3_ESM.pdf]

nature portfolio

Corresponding author(s): Asier Saez-Cirion

Last updated by author(s): Nov 24, 2023

Reporting Summary

Nature Portfolio wishes to improve the reproducibility of the work that we publish. This form provides structure for consistency and transparency in reporting. For further information on Nature Portfolio policies, see our Editorial Policies and the Editorial Policy Checklist.

Statistics

For all statistical analyses, confirm that the following items are present in the figure legend, table legend, main text, or Methods section.

n/a	Confirmed	
☐	☒	The exact sample size (*n*) for each experimental group/condition, given as a discrete number and unit of measurement
☐	☒	A statement on whether measurements were taken from distinct samples or whether the same sample was measured repeatedly
☐	☒	The statistical test(s) used AND whether they are one- or two-sided<br>*Only common tests should be described solely by name; describe more complex techniques in the Methods section.*
☐	☒	A description of all covariates tested
☐	☒	A description of any assumptions or corrections, such as tests of normality and adjustment for multiple comparisons
☐	☒	A full description of the statistical parameters including central tendency (e.g. means) or other basic estimates (e.g. regression coefficient) AND variation (e.g. standard deviation) or associated estimates of uncertainty (e.g. confidence intervals)
☐	☒	For null hypothesis testing, the test statistic (e.g. *F*, *t*, *r*) with confidence intervals, effect sizes, degrees of freedom and *P* value noted<br>*Give P values as exact values whenever suitable.*
☒	☐	For Bayesian analysis, information on the choice of priors and Markov chain Monte Carlo settings
☒	☐	For hierarchical and complex designs, identification of the appropriate level for tests and full reporting of outcomes
☐	☒	Estimates of effect sizes (e.g. Cohen's *d*, Pearson's *r*), indicating how they were calculated

Our web collection on statistics for biologists contains articles on many of the points above.

Software and code

Policy information about availability of computer code

Data collection	Flow cytometry data was collected with FlowJo software version 10 (Tree Star Inc.).Data storage was ensured by the BaTLab laboratory management system of IDMIT and data base interrogation and data visualization were performed using Tableau version 2021.3 (Tableau Software).
Data analysis	Graphs and statistical analyses were performed using Prism version 9.2.0 (GraphPad Software) or R version 4.2.2 (http://www.R-project.org). for flow cytometry data dimensionality reduction was performed using the uniform manifold approximation and projection (UMAP) algorithm (FlowJo plugin v3.1), and PhenoGraph was used for unsupervised clustering using default parameters (K=30).

For manuscripts utilizing custom algorithms or software that are central to the research but not yet described in published literature, software must be made available to editors and reviewers. We strongly encourage code deposition in a community repository (e.g. GitHub). See the Nature Portfolio guidelines for submitting code & software for further information.

Data

Policy information about availability of data

All manuscripts must include a data availability statement. This statement should provide the following information, where applicable:

- Accession codes, unique identifiers, or web links for publicly available datasets
- A description of any restrictions on data availability
- For clinical datasets or third party data, please ensure that the statement adheres to our policy

The data that support the findings of this study are available from the corresponding author Asier Saez-Cirion (asier. saez-cirion@pasteur.fr), upon reasonable

request.

Research involving human participants, their data, or biological material

Policy information about studies with human participants or human data. See also policy information about sex, gender (identity/presentation), and sexual orientation and race, ethnicity and racism.

Reporting on sex and gender	NA
Reporting on race, ethnicity, or other socially relevant groupings	NA
Population characteristics	NA
Recruitment	NA
Ethics oversight	NA

Note that full information on the approval of the study protocol must also be provided in the manuscript.

Field-specific reporting

Please select the one below that is the best fit for your research. If you are not sure, read the appropriate sections before making your selection.

[x] Life sciences [ ] Behavioural & social sciences [ ] Ecological, evolutionary & environmental sciences

For a reference copy of the document with all sections, see nature.com/documents/nr-reporting-summary-flat.pdf

Life sciences study design

All studies must disclose on these points even when the disclosure is negative.

Sample size	The sample size determined in the pVISCONTI study followed ethical recommendations and good practices for the use of non-human primates in biomedical research. Sample size were defined in order to assume a statistical analyses with a level of significance of 5% ($\alpha = 0.05$), a target power of 80% ($1 - \beta = 0.8$) and an effect size of 1.6 with the objective to use non-parametric tests (Kruskal-Wallis, Wilcoxon rank and Mann & Whitney tests) for group comparisons, with Dunn-Bonferroni correction) to adjust p-values when multiple comparisons are performed.
Data exclusions	One animal (CB296A) in the W4-treated group presented an extremely severe acute infection and reached the endpoint requiring euthanasia before ART initiation and one animal (BA979I) from the W24-treated group controlled viremia below 400 copies/mL before ART initiation. Their characteteristics and individual plasma viremia are presented in Table S1 and Figure S7 but they were excluded from further analyses. This is described in the Methods section.
Replication	The pVISCONTI study was performed in two independent experimental phases, each one including 6 W4- and 6 W24-treated macaques that were infected, treated, and monitored in parallel with reproducible results.
Randomization	Animals were allocated to each group to closely match in terms of age, weight, and genotype.<br>Sample collection and analyses were performed in random order
Blinding	The investigators were not blinded as per study design: sampling calendar was adapted to the delay to treatment initiation/treatment interruption for the different groups

Reporting for specific materials, systems and methods

We require information from authors about some types of materials, experimental systems and methods used in many studies. Here, indicate whether each material, system or method listed is relevant to your study. If you are not sure if a list item applies to your research, read the appropriate section before selecting a response.

Materials & experimental systems

n/a	Involved in the study	
[ ]	[x]	Antibodies
[x]	[ ]	Eukaryotic cell lines
[x]	[ ]	Palaeontology and archaeology
[ ]	[x]	Animals and other organisms
[x]	[ ]	Clinical data
[x]	[ ]	Dual use research of concern
[x]	[ ]	Plants

Methods

n/a	Involved in the study	
[x]	[ ]	ChIP-seq
[ ]	[x]	Flow cytometry
[x]	[ ]	MRI-based neuroimaging

Antibodies

Antibodies used	The following antibodies were used: anti-CD3–AF700 (clone SP34-2, BD Biosciences), Cross reactivity: Technical Datasheet, Dilution: 1/30. anti-CD4–PerCP-Cy5.5 (clone L200, BD Biosciences), Cross reactivity: Technical Datasheet, Dilution: 1/30. anti-CD8–APC-Cy7 (clone RPA-T8, BD Biosciences), Cross reactivity: Technical Datasheet, Dilution: 1/30. anti-CD38–FITC (clone AT-1, StemCell Technologies), Cross reactivity: Technical Datasheet, Dilution: 1/15. anti-HLA-DR–V450 (clone G46-6, BD Biosciences), Cross reactivity: Technical Datasheet, Dilution: 1/30. anti-CD45RA–PE Cy7 (clone 5H9, BD Biosciences), Cross reactivity: Technical Datasheet, Dilution: 1/30. anti-CCR7–PE-Dazzle594 (clone G043H7, Biolegend), Cross reactivity: Technical Datasheet, Dilution: 1/10. anti-CD27–PE (clone M-T271, BD Biosciences), Cross reactivity: Technical Datasheet, Dilution: 1/7. anti-Ki-67– AF647 (clone B56, BD Biosciences), Cross reactivity: laboratory control (FMO vs Positive) & Passaes et al 2020, Dilution: 1/30. anti-CD127–BUV496 (clone HIL-7R-M21, BD Biosciences), Cross reactivity: laboratory control (FMO vs Positive) & Passaes et al 2020, Dilution: 1/20. anti-CD107a–BV786 (clone H4A3, BD Biosciences), Cross reactivity: NHP Reagents Database (https://www.nhpreagents.org/ReactivityDatabase), Dilution: 1/20. anti-TNFα–BUV395 (clone MAb11, BD Biosciences), Cross reactivity: Technical Datasheet, Dilution: 1/20. anti-IFNγ–BV605 (clone B27, BD Biosciences), Cross reactivity: NHP Reagents Database (https://www.nhpreagents.org/ReactivityDatabase), Dilution: 1/20. anti-IL2–BUV737 (clone MQ1-17H12, BD Biosciences), Cross reactivity: Technical Datasheet, Dilution: 1/20. anti-phospho S6 S235/236–Pacific blue (clone D57.2.2E, Cell Signaling), Cross reactivity: Technical Datasheet, Dilution: 1/25. and anti-phospho Akt Ser473–AF647 (clone D9E, Cell Signaling), Cross reactivity: Technical Datasheet, Dilution: 1/25. anti-TCF-7/TCF-1–AF488 (clone S33-966, BD Biosciences), Cross reactivity: laboratory control (FMO vs Positive) & Passaes et al 2020, Dilution: 1/10. anti-CD279–BV421 (clone EH12.2H7, Biolegend), Cross reactivity: Technical Datasheet, Dilution: 1/10. anti-CD39–BV785 (clone A1, Biolegend), Cross reactivity: Technical Datasheet, Dilution: 1/10.
Validation	Manufacturer's Specifications: We reviewed the manufacturer's specifications and technical data sheets for each antibody. This information included details such as clone names, isotypes, recommended dilutions, and references. Understanding these specifications was essential for proper antibody usage.<br>Positive and Negative Controls: To validate antibody performance, we used positive and negative controls. Positive controls included cells known to express the target antigen, while negative controls were cells lacking the antigen.<br>Fluorescence Minus One (FMO) Controls: FMO controls were utilized to assess background fluorescence.<br>Non-Human Primate (NHP) Reagent Controls: NHP reagent controls were used to ensure the compatibility and specificity of the antibodies in the primate model.

Animals and other research organisms

Policy information about studies involving animals; ARRIVE guidelines recommended for reporting animal research, and Sex and Gender in Research

Laboratory animals	Cynomolgus macaques, imported from Mauritius (median age = 4.8 years at inclusion, IQR = 3.9-7.2).
Wild animals	No wild animals were used in the study
Reporting on sex	In the present study only male macaques were used, but the conclusions are not sex-based.
Field-collected samples	No field collected samples were used in the study
Ethics oversight	CyMs were imported from Mauritius and housed in facilities at the Infectious Disease Models and Innovative Therapies (IDMIT) center (CEA site at Fontenay-aux-Roses, France). All non-human primate studies at IDMIT are conducted in accordance with French National Regulations under the supervision of National Veterinary Inspectors (CEA Permit Number D92-032-02). IDMIT complies with the Standards for Human Care and Use of Laboratory Animals of the Office for Laboratory Animal Welfare under Assurance Number #A5826-01 and F20-00448. All experimental procedures were conducted according to European Directive 2010/63 (Recommendation Number 9). The pVISCONTI study was approved and accredited under the statement A15 035 from the "Comité d'Ethique en Expérimentation Animale du CEA", registered and authorized under Number 2453-2015102713323361v3 by the French Ministry of Education and Research. CyMs were studied with veterinary guidance, housed in adjoining individual cages allowing social interactions, and maintained under controlled conditions with respect to humidity, temperature, and light. Water was available ad libitum. Animals were monitored and fed with commercial monkey chow, vegetables and fruits once or twice daily by trained personnel. Environmental enrichment was provided in the form of toys, novel foodstuffs, and music under the supervision of IDMIT Animal Welfare Body. Experimental procedures (animal handling, viral inoculations, and samplings) were conducted after sedation with ketamine chlorhydrate (Imalgene 1000®, 10 mg/kg, intravenously (i.v.), Merial). Tissues were collected during follow up and at necropsy. Animals were euthanized after ketamine chlorhydrate sedation followed by a bolus of sodium pentobarbital (Doléthal, 180 mg/kg, i.v., Laboratoire Vetoquinol).

Note that full information on the approval of the study protocol must also be provided in the manuscript.

Flow Cytometry

Plots

Confirm that:

- [x] The axis labels state the marker and fluorochrome used (e.g. CD4-FITC).
- [x] The axis scales are clearly visible. Include numbers along axes only for bottom left plot of group (a 'group' is an analysis of identical markers).
- [x] All plots are contour plots with outliers or pseudocolor plots.
- [x] A numerical value for number of cells or percentage (with statistics) is provided.

Methodology

Sample preparation

PBMCs and tissue samples were analyzed by Flow Cytometry in the present study. A detailed protocol for sample processing is indicated in the methods section, as follows:

Blood collection and processing

Peripheral blood was collected by venous puncture into Vacutainer Plus Plastic K3EDTA Tubes or Vacutainer CPT Mononuclear Cell Preparation Tubes with Sodium Heparin (BD Biosciences). Complete blood counts were monitored at all time points from the Vacutainer Plus Plastic K3EDTA Tubes. Plasma was isolated either from Vacutainer Plus Plastic K3EDTA Tubes by centrifugation for 10 min at 1,500 rpm, or from Vacutainer CPT Mononuclear Cell Preparation Tubes after centrifugation for 40 min at 3,000 rpm, and stored at −80°C. Peripheral blood mononuclear cells (PBMCs) were isolated from Vacutainer CPT Mononuclear Cell Preparation Tubes with Sodium Heparin according to manufacturer's instructions (BD Biosciences), and red blood cells were lysed in Ammonium-Chloride-Potassium (ACK) buffer (0.15 M NH4Cl, 10 mM KHCO3, 0.1 mM EDTA, pH 7.4).

Tissue collection and processing

Axillary or inguinal lymph nodes (peripheral PLNs), bone marrow samples (BM), and broncho-alveolar lavages (BAL) were collected longitudinally (Figure S1). In addition, spleen, mesenteric lymph nodes (MLNs), colon and liver were collected at necropsy. Tissue samples were collected in RPMI medium at 2–8°C. Lymph node cells were isolated into RPMI medium via mechanical disruption using a gentleMACS Dissociator (Miltenyi Biotec). The cell suspension was filtered (70 µm), and red blood cells were lysed in ACK. Bone marrow cells were purified using Lymphocyte Separation Medium (Eurobio Scientific) diluted to 90% in DPBS, centrifuged for 20 min at 350 g, and separated from red cells in ACK. Spleen cells were processed via mechanical disruption in RPMI medium using a gentleMACS Dissociator (Miltenyi Biotec), purified as described for BM cells, and separated from red cells in ACK. Colonic lymphocytes were obtained from mucosa taken from approximately 10 cm of tissue. Colonic tissue was washed extensively in PBS and R10 medium (RPMI medium supplemented with 10% fetal calf serum and penicillin/streptomycin), and then digested for 45 min with collagenase II prior to mechanical disruption. Lymphocytes were isolated over a Percoll 67/44 gradient (Sigma-Aldrich). Liver tissue was mechanically disrupted using the gentleMACS Dissociator (Miltenyi Biotec) and cell suspension was sequentially filtered (300µm > 100µm > 70µm), then lymphocytes were obtained over an OptiPrep gradient (Sigma-Aldrich). T cell activation, proliferation and exhaustion phenotyping and the measurements of SIV-suppressive activity ex vivo were performed using freshly isolated cells. T cell CFSE proliferation assay and cytokine intracellular staining were performed using viable cells frozen at −196°C in DMSO/FCS.

Instrument

Data were acquired using either a LSRII flow cytometer (BD Biosciences) or a Fortessa flow cytometer (BD Biosciences).

Software

Data was analyzed with FlowJo software version 10 (Tree Star Inc.).

Cell population abundance

n/a

Gating strategy

The general gating strategy used in the analyses explored in the current study was as follows:
singlets (FSC-A/FSC-H) > morphology > (FSC-A/SSC-A) > live cells (SSC-A/live-dead marker) > CD3+ population (SSC-A/CD3-Alexa fluor 700) > CD4/CD8 (CD4-PerCP Cy5.5/CD8-APCCy7).
The differentiation status of T cells were defined as follows: Naïve: CD45RA+CD27+CCR7+; central memory (CM): CD45RA-CD27+CCR7+; transitional memory (TM): CD45RA-CD27+CCR7-; effector memory (EM): CD45RA-CD27-CCR7-; effector: CD45RA+CD27-CCR7-.
Activation (CD38/HLADR, proliferation (Ki-67), cytokine production and the levels of phospo S6 and phospho Akt proteins were evaluated in T cells.
Negative and positive signals were determined with the help of a FMO control and/or upon Concanavalin-A stimutated or non-stimulated conditions.

- [x] Tick this box to confirm that a figure exemplifying the gating strategy is provided in the Supplementary Information.
